# Exploring Nursing Students’ Experiences of the COVID-19 Period at a Public Nursing College in the Eastern Cape Province of South Africa

**DOI:** 10.3390/ijerph23030395

**Published:** 2026-03-20

**Authors:** Ntombedinga Tilly Goso, Ntiyiso Vinny Khosa, Malwande Shooster Mgilane, Thokoe Vincent Makola, Nomfuneko Sithole

**Affiliations:** 1 School of Public Health, Faculty of Medicine and Health Sciences, Walter Sisulu University, Mthatha 5117, South Africa; 2 WSU Institute for Clinical Governance and Healthcare Administration, Faculty of Medicine and Health Sciences, Walter Sisulu University, East London 5257, South Africa; 3 Global Centre for Human Resources for Health Intelligence, Faculty of Medicine and Health Sciences, Walter Sisulu University, East London 5257, South Africa

**Keywords:** COVID-19 pandemic, student well-being, nursing education, clinical training disruptions, South Africa

## Abstract

**Highlights:**

**Public health relevance—How does this work relate to a public health issue?**

- The research explored how the disruptions caused by COVID-19 affected the education, well-being, and professional development of nursing students, who represent a critical component of the future health workforce. This study contributes to a deeper understanding of how education and training are affected by public health emergencies, particularly in resource-constrained settings.

**Public health significance—Why is this work of significance to public health?**

- This study contributes to the limited body of observed evidence detailing how disruptions to nursing education can extend beyond academic challenges to influence health workforce preparedness, professional confidence, and long-term health system capacity in rural provinces such as the Eastern Cape Province.

**Public health implications—What are the key implications or messages for practitioners, policy makers and/or researchers in public health?**

- This research has important implications for public health policy, health workforce planning, and nursing education reform. The study highlights the need to strengthen health workforce education systems, importance of investing in digital infrastructure and blended learning systems and the need to integrate comprehensive mental health and wellness support services for nursing students.

**Abstract:**

**Introduction:** The COVID-19 pandemic significantly disrupted the functioning of the health system, including nursing education, particularly within resource-constrained contexts such as in South Africa. This study explored the lived experiences of nursing students during the COVID-19 period at Lilitha College of Nursing, a public nursing college operating across multiple urban and rural campuses in the Eastern Cape Province of South Africa. **Methodology:** A qualitative phenomenological design was employed, guided by the Dimensions of Wellness Framework. Purposive sampling was used to recruit a diverse cohort of nursing students who were registered during the period 2020–2022. Semi-structured, one-on-one interviews were conducted with 20 participants between 1 and 31 October 2025, until data saturation was attained. Interviews were audio-recorded, transcribed, translated verbatim, and analysed manually using the six phases of thematic analysis. **Results:** The findings revealed that the COVID-19 pandemic severely affected nursing students’ academic progress, mental and physical health, clinical training, and overall well-being, revealing institutional unpreparedness and gaps in support during crisis conditions. **Conclusions:** The study highlights the need for fair, holistic, and crisis-ready support systems to protect nursing students’ well-being and learning during future emergencies.

## 1. Introduction

Pandemic periods have historically posed significant challenges to societies, health systems, and educational institutions worldwide [1]. Such crises are typically characterised by widespread uncertainty, increased morbidity and mortality, and profound disruptions to social and economic life. Pandemics often necessitate extraordinary societal responses, including stringent public health measures to limit disease transmission [2]. Vital disease control measures, such as quarantine, movement restrictions, and the reorganisation of essential services, often generate unintended consequences, particularly for vulnerable populations and sectors that rely heavily on face-to-face human interaction, such as education and healthcare training [3]. The global COVID-19 pandemic exemplified these challenges, causing unprecedented disruptions across health systems, educational institutions, and communities worldwide. Within the education sector, the pandemic forced an abrupt transition from traditional classroom-based learning and bedside clinical instruction to emergency remote learning modalities [4]. For nursing students, whose education depends heavily on experiential and clinical training, these changes significantly altered the learning environment. Students experienced interruptions to clinical placements, reduced opportunities for hands-on skills development, and uncertainty regarding academic progression and competency attainment. While these disruptions were experienced globally, their impact was particularly pronounced in low- and middle-income countries such as South Africa, where structural inequalities, limited digital infrastructure, and socioeconomic disparities shaped students’ ability to adapt to emergency remote learning and other pandemic-related changes [5,6,7,8].

In the South African context, the pandemic amplified pre-existing challenges within higher education and nursing training systems [9]. Public nursing colleges were required to rapidly adapt curricula, teaching modalities, and clinical training arrangements in response to fluctuating lockdown regulations and infection control protocols. These adaptations occurred within a system already grappling with resource limitations and unequal access to educational technologies. Consequently, many nursing students encountered barriers, including limited access to reliable internet connectivity, a lack of digital devices, and inadequate study environments at home [10]. These challenges were particularly evident in the Eastern Cape Province, a province characterised by widespread poverty, rurality, high unemployment, and an overburdened health system [11]. In such resource-constrained contexts, the shift to remote learning and disruptions to clinical training may have had lasting consequences for students’ academic engagement, skills development, and overall educational experience. Although considerable attention during the pandemic focused on ensuring the continuity of teaching and learning [12], the longer-term implications of these disruptions are increasingly becoming evident in the post-pandemic period [13]. Nursing students who trained during the COVID-19 crisis may have experienced gaps in clinical exposure, reduced opportunities for mentorship, and increased academic and psychological strain. In resource-constrained provinces such as the Eastern Cape, these impacts may persist beyond the immediate crisis, potentially influencing students’ confidence, preparedness for professional practice, and transition into the health workforce. Understanding these experiences is therefore essential for informing post-pandemic recovery strategies that strengthen nursing education systems, particularly in contexts where health services depend heavily on newly qualified nurses to sustain service delivery.

South Africa’s national strategies aimed at strengthening nursing and midwifery education, workforce development, and quality assurance have been implemented through policies such as the National Health Act, the National Development Plan 2030, and the Human Resources for Health Strategy for South Africa [14,15,16]. These frameworks emphasise the importance of experiential learning, flexible curriculum delivery, and institutional preparedness to maintain training continuity during emergencies [17]. However, the effectiveness of such policies in addressing lived realities during crisis periods remains insufficiently explored, particularly in historically under-resourced provinces. Nursing students represent a critical component of the future health workforce pipeline [18]. In South Africa, nurses constitute the largest segment of the health workforce, estimated at 56% [19], and play a pivotal role in achieving national health priorities, including strengthening primary healthcare, universal health coverage, and equitable service delivery. Disruptions to nursing education during health emergencies therefore have implications not only for students’ individual learning trajectories but also for the broader sustainability and resilience of the health system. In resource-constrained settings like the Eastern Cape Province, where health facilities already experience workforce shortages [11], the preparedness and competence of newly qualified nurses are particularly vital. Despite the central role of nursing students in sustaining the health workforce [20], there is limited evidence documenting their lived experiences during the COVID-19 period, particularly in public nursing colleges. Understanding how students navigated the academic, clinical, and psychosocial challenges of the pandemic is critical for identifying persistent gaps in training and for informing post-pandemic recovery and resilience planning in nursing education.

This study, therefore, aims to explore the experiences of nursing students during the COVID-19 pandemic at a public nursing college in the Eastern Cape Province of South Africa. By capturing students’ perspectives on the academic, clinical, and psychosocial challenges encountered during this time, the study seeks to generate evidence that can inform post-pandemic strengthening of nursing education, guide institutional preparedness for future health crises, and support the development of responsive training systems capable of sustaining the nursing workforce, particularly at Lilitha Nursing College.

## 2. Materials and Methods

### 2.1. Study Design and Conceptual Framework

**Figure 1:**
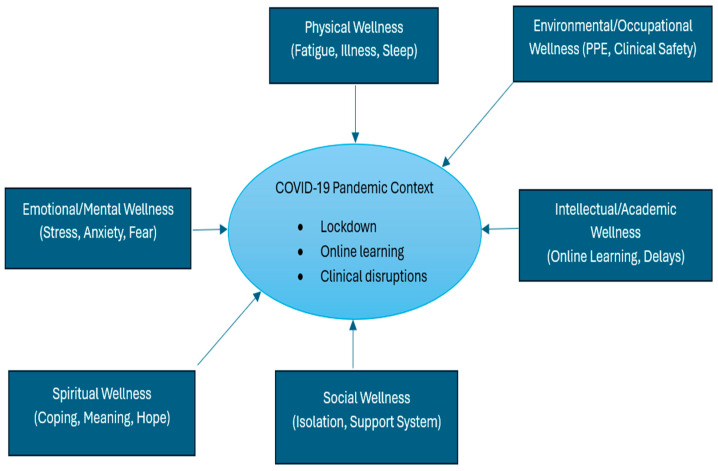
Conceptual framework illustrating the impact of the COVID-19 period on multiple dimensions of wellness among nursing students at a public nursing college in the Eastern Cape.

This study adopted a qualitative research methodology to explore the experiences of nursing students during the COVID-19 period at a public nursing college in the Eastern Cape Province of South Africa. The research design employed a qualitative phenomenological approach, primarily focusing on exploring participants’ lived experiences related to the phenomenon under study [21]. The researchers adhered to a bracketing principle of reflexivity, consciously setting aside their prior knowledge, assumptions, beliefs, and experiences about the phenomenon being studied [22]. The supervisor served as the bracketing interviewer using the study interview guide and interviewed the data collector. The interview results were presented and discussed at a meeting, and comments were made on the responses. The written reflection of the bracketing experience was added to the reflective journal and maintained throughout the study [22]. The study was further guided by the Dimensions of Wellness Framework (Figure 1), adopted in nursing education, which conceptualises the COVID-19 pandemic as a contextual stressor influencing multiple interrelated domains of nursing students’ well-being [23]. The Dimensions of Wellness Framework views health as a holistic and multidimensional construct encompassing physical, emotional, social, intellectual, spiritual, and occupational well-being [23]. The framework was particularly relevant for exploring nursing students’ experiences during the COVID-19 pandemic, as the pandemic disrupted multiple aspects of wellness simultaneously. The study applied the framework to examine how COVID-19 influenced nursing students’ physical and mental health within the broader context of their academic, social, and clinical environments.

### 2.2. Study Setting

The study was conducted at Lilitha College of Nursing, a public nursing college located in the Eastern Cape Province of South Africa. Lilitha College of Nursing is responsible for the education and training of professional nurses for the provincial public health sector [24]. The college is operated under the Eastern Cape Department of Health across multiple campuses located in both urban and rural settings, serving students from diverse socioeconomic backgrounds [25]. The campuses are in Port Elizabeth, East London, Queenstown, Mthatha, and Lusikisiki. Lilitha College plays a critical role in training nurses to staff public hospitals, clinics, and community health services across the Eastern Cape Province [26]. The Eastern Cape Province is predominantly rural and characterised by resource constraints, high unemployment, and a substantial burden of disease [11], factors that influence both healthcare delivery and nursing education.

### 2.3. Population and Sampling

The study population included all students registered at the college’s campuses during the COVID-19 pandemic period (2020, 2021, and 2022). The study involved registered final-year students. Participants who met the study criteria were purposively sampled to provide rich insights into their lived experiences during the COVID-19 period. A sample size of 15–20 participants was targeted until data saturation was reached. The included participants were registered nursing students aged 18 to 55 years at the time of the study, who were also registered during the COVID-19 period at Lilitha College of Nursing and provided informed consent for participation. The study excluded participants who were registered at the college during the COVID-19 period but were not registered at the time of the study.

### 2.4. Data Collection

Qualitative data were collected using a predefined interview guide consisting of demographic, experience, challenges, and support-related questions from 1 to 31 October 2025. The interview guide consisted of open-ended questions to facilitate discussion while allowing participants to narrate their experiences in their own words. Probing questions were used to clarify responses and explore emerging issues in greater depth. Interview appointments were scheduled with participants at each college campus. Before data collection commenced, participants were briefed on the study background and purpose, and the research process was explained. Each participant was provided with a study information sheet to further familiarize themselves with the study details and was informed that their participation was voluntary. Data were collected through semi-structured one-on-one interviews conducted in both the native language, IsiXhosa, and English. The interviews were conducted in a private and comfortable room to encourage open and honest discussion. During the interviews, participants completed and signed consent forms, indicating their permission to participate and to be audio-recorded. Data collection continued until data saturation was reached, at which point no new themes or insights emerged from subsequent interviews. Each interview was conducted in a language preferred by the participant, lasted approximately 30 to 45 min, and was audio-recorded. Data saturation was determined through ongoing preliminary analysis of data already collected, which identified and confirmed that no new information or insights emerged from subsequent interviews. This study achieved saturation after 20 interviews, aligning with established qualitative research practices.

### 2.5. Trustworthiness of the Data

Trustworthiness is the foundation for the accuracy of qualitative research findings. In this study, the researcher ensured that it was grounded in the following trustworthiness criteria: credibility, dependability, confirmability, transferability, and authenticity.

**Credibility**

Credibility was achieved by adopting the most appropriate sampling strategy to identify and recruit participants who met the predetermined inclusion criteria. In this study, this strategy was characterised by the adoption of appropriate data collection methods, including open-ended interviews to capture authentic experiences.

**Transferability**

Transferability refers to the extent to which the findings may be applicable in similar contexts. To support transferability, the study provided descriptions of the research setting, participant inclusion criteria, and contextual factors specific to the nursing college and the broader Eastern Cape Province. This detailed description allows readers to determine whether the findings may be relevant to other similar educational or resource-constrained settings.

**Dependability**

This concerns the consistency and reliability of the research process over time. This was achieved by maintaining a detailed audit trail documenting all stages of the research process, including methodological decisions, interview procedures, and data analysis steps. This allowed others involved in the research to follow the process. The following strategies were also adopted: (i) providing research protocols to the research team, and (ii) using data collection techniques such as the use of an audio recording to ensure the reliability of the data collected. The researcher also applied the code-recode method, in which the same data were coded twice by two individuals involved in the study, with a two-week gap between the coding processes. The two coded results were then compared for consistency.

**Confirmability**

This ensures that the findings are shaped by the participants’ experiences rather than the researcher’s bias. To enhance confirmability, the researchers applied reflexivity and bracketing and maintained a reflexive journal throughout the study. This helped ensure that the interviews and their interpretations were grounded in the data collected rather than the researchers’ preconceived views. Additionally, a comprehensive literature review was undertaken to compare similarities and differences and ensure that the results obtained were supported by the literature.

**Authenticity**

In this study, authenticity was defined as the researcher’s capacity to communicate respondents’ feelings, experiences, and emotions, as explained by [27]. In this study, respondents’ quotations were used in the narratives.

### 2.6. Data Processing and Analysis

Data captured in the audiotape recordings were transcribed verbatim and translated into English exactly as spoken. The data were processed manually, without any software. The six phases of thematic analysis were adopted to analyse qualitative data [28]. The analysis process began with familiarization, during which audio recordings were repeatedly listened to, and the transcripts were reviewed to ensure data accuracy and completeness. Initial codes were then generated, in which the data were systematically categorized and meaningfully organized. The next step involved examining and clustering codes into broader thematic categories. During the theme review stage, the researcher ensured that the emerging themes accurately reflected the data. Subsequently, the themes were named, refined, clearly articulated, and distinguished to capture specific aspects of the data. In the final report phase, the study’s findings were synthesized and evaluated, highlighting key lessons learned in the essence of the phenomenon under investigation.

### 2.7. Ethics Approval

Ethical clearance to conduct the study was granted by the Walter Sisulu University Health Science Faculty Ethics Committee (Ethics approval number: 195/2025). For data collection, permission was granted by the Eastern Cape Department of Health Research Ethics Committee and by the management of Lilitha College of Nursing, as gatekeepers of the research sites.

## 3. Results

### 3.1. Demographic Profile of Participants

A total of 20 nursing students participated in the study. Participants were recruited from five Lilitha Nursing College campuses (Queenstown, East London, Port Elizabeth, Mthatha, and Lusikisiki) in the Eastern Cape Province. Participants included both males (*n* = 8) and females (*n* = 12), with ages ranging from 27 to 51 years, as shown in Table 1. All participants were fourth-year nursing students. Participants were enrolled across all campuses of the college, with a larger proportion coming from Queenstown Campus, Mthatha Campus, and Lusikisiki Campus. Representation from multiple campuses enabled exploration of diverse academic, psychological, and physical health experiences related to the COVID-19 pandemic.

Themes and subthemes are summarised in a thematic matrix form in Table 2.

### 3.2. Theme 1: Experiences During the COVID-19 Pandemic

#### 3.2.1. Disruption to Academic Progress and Learning

Participants reported significant disruption to their academic progress following the onset of the COVID-19 pandemic. Contact classes were abruptly suspended, academic calendars were delayed, and teaching shifted to remote platforms. Most participants indicated that teaching and communication were conducted primarily via WhatsApp, which they perceived as inadequate for nursing education.

*“We started school in February, and then in March, we were told there was COVID-19 and we had to go home.”*

(Participant 4)

Limited interaction with lecturers and insufficient academic feedback were frequently reported. Participants described challenges in understanding course content and keeping up with academic requirements. Upon returning to campus, participants reported that lecturers attempted to cover missed content within limited time frames.

*“It delayed student-lecturer contact, which became a major challenge. Most of the time, we communicated* via *WhatsApp, which was the only contact we had with lecturers”.*

(Participant 6)

Some participants reported failing modules or repeating academic years due to the disruptions.

*“Academically, it affected me because I failed that year. I failed that year, ma’am.”*

(Participant 2)

Barriers to effective communication and learning included limited student–lecturer interaction, poor network connectivity and insufficient digital skills.

*“You would get a voice note or a WhatsApp message, which is different from a proper lecture.”*

(Participant 19)

*“Sometimes we did not have data, sometimes airtime, so we stayed in that situation.”*

(Participant 12)

#### 3.2.2. Psychological and Emotional Distress

Psychological and emotional distress emerged strongly across all interviews. Participants described feelings of persistent fear, anxiety, and uncertainty related to infection, death, and the future. Exposure to high COVID-19-related mortality rates and continuous media coverage intensified emotional strain. Several participants reported experiencing personal losses, including the death of parents or relatives, which resulted in grief, self-diagnosed depression, and long-term emotional trauma. Lockdown-related isolation further exacerbated emotional distress, leading to loneliness, helplessness, and emotional exhaustion. These experiences negatively impacted both academic engagement and personal well-being.

*“I feared being infected and spreading it to my family. I also feared losing my mother, my father, or one of my siblings. I also feared that I might die.”*

(Participant 18)

Lockdown measures and prolonged isolation were described as stressful experiences.

*“Mentally, yes. I was not coping at home. Staying at home doing nothing was stressful. That is how it affected me mentally.”*

(Participant 18)

#### 3.2.3. Impact on Personal Life and Social Well-Being

The COVID-19 pandemic was reported to have significantly altered the participants’ personal lives. Participants commonly reported increased family responsibilities, changes in daily routines, and reduced social interaction. Some participants reported caring for ill family members during lockdown.

*“My parents were also sick with COVID, and I am the firstborn, so I had to attend to everything. I had to be everywhere at the same time. It was a big challenge; I will not lie.”*

(Participant 10)

Social isolation from peers and reduced access to support networks were also reported. Many students felt disconnected from their academic communities, which further intensified feelings of loneliness and stress.

*“We could not access social workers or have loved ones comfort us. We were on our own.”*

(Participant 1)

*“We were not seeing each other. Everyone was living in their rooms, and each person was studying alone.”*

(Participant 15)

### 3.3. Theme 2: Challenges Experienced During and Post-COVID-19

#### 3.3.1. Mental Health Challenges

Participants reported experiencing ongoing mental health challenges during and after the pandemic, which included stress, anxiety, depression, and emotional breakdowns. Some participants reported formal diagnoses with mental health conditions, while others reported persistent self-realised psychological symptoms, such as those of panic attacks and trauma-related symptoms.

*“I was diagnosed with major Depression based on losing my father due to COVID-19. I am still taking antidepressants.”*

(Participant 20)

*“Psychologically, it….it was hard to adapt. You could not visit any other places, and you stayed home in one place. It affected us mentally.”*

(Participant 1)

*“I was never okay after that. When I tried to study, my mind was somewhere else. I had seizures about three times during the night.”*

(Participant 7)

Participants commonly reported fear as one of the debilitating feelings during the pandemic; fear of infection, death, academic failure, poor professional competence, and the inability to complete training.

*“My biggest fear was dying.”*

(Participant 5)

*“There was a fear of failing.”*

(Participant 17)

*“I was worried I would not finish the course because I love nursing.”*

(Participant 9)

*“My fear was getting infected with COVID-19 and not achieving academically because we were not getting enough education.”*

(Participant 3)

#### 3.3.2. Physical Health Challenges

Participants reported experiencing physical health challenges during the pandemic, including fatigue, body pains, loss of appetite, and general weakness. Some reported COVID-19 infection or adverse reactions following vaccination, which temporarily affected their physical functioning.

*“Yes. There were physical challenges in the sense that one day I had to take the job, the first one. My body ached”*

(Participant 2)

*“Yes, it did affect me because I was diagnosed with COVID-19 myself. I was coughing, having a fever, losing weight, vomiting, and waking up in the middle of the night because I could not breathe”*

(Participant 15)

#### 3.3.3. Challenges in Clinical and Hands-On Training

Clinical training was reported to have been significantly disrupted during the pandemic. Participants reported limited hands-on practice, reduced opportunities to develop clinical skills, and anxiety upon returning to clinical settings. Fear of infection and exposure to patient deaths, and concerns of inadequate preparedness were commonly reported.

*“No, I had no hands-on experience as we were asked to go home.”*

(Participant 3)

*“Yes, of course. After we were recalled by the school, we were allocated to the wards in my institution. It was a tough situation, people were dying, people were sick.”*

(Participant 5)

### 3.4. Theme 3: Support Services

#### Institutional Support for Mental and Physical Health

Most participants reported limited institutional support during the pandemic. Counselling services, psychologists, and social workers were reported as either unavailable or inconsistently accessible across campuses. Some participants reported that support was informal and depended on individual lecturers, and this contributed to feelings of neglect and increased emotional distress among students.

*“You just tell an individual lecturer, but there is no official emotional support system. There is also no office that deals with wellness.”*

(Participant 5)

*“I have never heard of a psychologist specifically for the school.”*

(Participant 8)

*“The only accessible support we have is from Lecturers.”*

(Participant 10)

*“We had nowhere to go if we faced a problem. Ideally, we could share our challenges with a social worker or psychologist, but we were on our own.”*

(Participant 1)

### 3.5. Theme 4: Participants’ Recommendations for Future Preparedness

Participants provided recommendations to improve institutional preparedness and student support. The recommendations included the appointment of permanent psychologists and social workers, the establishment of campus wellness centers, the provision of laptops and data, improved communication systems, and the development of emergency preparedness plans.

*“The College should create departments like social development with social workers and psychologists. Having psychologists would help a lot.”*

(Participant 1)

*“We need psychologists in the school so that we can get counselling when we face problems or challenges.”*

(Participant 13)

## 4. Discussion

Participants in this study reflect a diverse cohort of nursing students drawn from multiple campuses of Lilitha Nursing College in the Eastern Cape Province, thereby strengthening the credibility and richness of the findings. This diversity enabled the exploration of varied institutional contexts and learning environments, which is particularly important when examining experiences during COVID-19, as its impact was not uniform across settings. The gender distribution, with a higher proportion of female participants (*n* = 12) than male participants (*n* = 8), aligns with the traditionally female-dominated nature of the nursing profession in both South Africa and globally [29]. Age diversity also suggests differences in life responsibilities, coping strategies, and resilience levels, which are likely to have shaped students’ experiences with academic, psychological, and physical health challenges during the pandemic. Mature students, for example, may have faced compounded pressures such as family caregiving responsibilities, financial constraints, and heightened health concerns, intensifying the impact of pandemic-related disruptions [30]. The findings demonstrate that the COVID-19 pandemic had a significant and multidimensional impact on nursing students’ academic, psychological, and social experiences. Disruptions to contact teaching and academic schedules, coupled with the rapid transition to remote learning via digital platforms such as WhatsApp, exposed institutional limitations in facilitating emergency online education [31]. Although the platform enabled basic communication, it was widely perceived as inadequate for delivering complex, skills-based nursing content, resulting in reduced engagement, limited feedback, and constrained opportunities for academic discussion and support [32]. These findings reflect broader challenges in nursing and health sciences education during the pandemic, where institutions struggled to rapidly transition to structured digital learning systems.

Importantly, these findings have implications beyond the pandemic period and highlight emerging and future challenges in nursing education. The reliance on informal digital tools such as WhatsApp demonstrates the urgent need for institutional investment in robust digital learning infrastructure, including learning management systems, faculty training in online teaching, and equitable access to technological resources for students. Without such investment, future disruptions, whether caused by pandemics, related disasters, or other systemic shocks, may once again compromise educational continuity. For resource-constrained institutions such as Lilitha Nursing College in the Eastern Cape Province, strengthening digital readiness must therefore become a critical priority for building resilient education systems. Digital inequities also emerged as a major barrier to effective learning. Limited access to mobile data, unreliable connectivity, and inadequate technological skills hindered students’ consistent participation in remote learning. These challenges are consistent with evidence from other low- and middle-income settings where unequal access to digital resources amplified pre-existing educational inequalities during the pandemic [33]. For students whose training depends on structured academic instruction and clinical integration, such barriers undermine academic progression. Efforts to recover lost teaching time through accelerated curricula were reported to contribute to academic overload, increased stress, and poor academic outcomes, including module failures and the repetition of academic years. These findings highlight a critical challenge for future nursing education: balancing curriculum recovery with student well-being and learning quality, particularly when institutions must respond rapidly to educational disruptions [34].

Psychological and emotional distress was another prominent feature of students’ experiences during the pandemic. Fear of infection, anxiety about transmitting the virus to family members, uncertainty about death and the future, and experiences of bereavement contributed to grief, depression, and lasting psychological trauma [35]. Lockdown-related social isolation and the disruption of peer and family support networks further intensified emotional strain. These findings reinforce the strong relationship between mental health and academic functioning [36] and highlight a critical gap in student wellness services within this nursing education institution. From a future-oriented perspective, the findings underscore the importance of integrating structured mental health and wellness services into nursing education systems. Mental health support should not be viewed solely as an emergency intervention during crises but rather as a core component of student support and professional development. The COVID-19 pandemic exposed broader weaknesses in the health and wellness services available to the health workforce, including nursing students, which should ideally form part of occupational health services within provincial health departments. Ensuring that the mental health and well-being of the health workforce are addressed within future health workforce strategies will therefore be critical for strengthening both education systems and health system resilience.

Participants also reported persistent physical health challenges during and after the pandemic, including fatigue, sleep disturbances, and COVID-19-related illnesses. These physical health challenges were closely intertwined with psychological distress, reinforcing the need for holistic student support strategies that address both mental and physical well-being [37]. For nursing education institutions, this implies adopting more integrated wellness frameworks that recognise the complex relationship between student health, academic performance, and professional preparedness. Clinical training disruptions emerged as one of the most significant consequences of the pandemic. The suspension of or reduction in placements limited opportunities for hands-on skills development and undermined students’ confidence and perceived readiness for professional practice [38]. When students returned to clinical environments, many encountered overstretched healthcare facilities, high patient mortality, and emotionally distressing working conditions. These experiences highlight a critical lesson for future nursing education: the need to diversify clinical training approaches, including the greater use of simulation-based learning, structured mentorship programmes, and flexible clinical placement models that can be adapted during health emergencies.

Another important implication relates to institutional preparedness and policy implementation. Participants reported limited availability and accessibility of institutional support services during the pandemic, reflecting a gap between educational policy intentions and practical implementation during crisis conditions [39]. While national and international nursing education policies emphasise the importance of student support services [40], the findings suggest that these policies were not fully operationalised to address the unprecedented psychosocial and academic demands created by the pandemic. This indicates a need for clear institutional emergency preparedness frameworks that integrate academic continuity planning, mental health services, and student support mechanisms. In the absence of formal support systems, students often relied on individual lecturers for emotional and academic support. While this informal support was valued, it was inconsistent and unsustainable. These findings highlight the need for coordinated, institution-wide support structures rather than reliance on individual efforts by academic staff. Participants offered several forward-looking recommendations to strengthen nursing education systems in preparation for future crises. These included establishing campus-based wellness centres, which [41] also viewed as important, improving institutional communication, and clearly defining emergency preparedness plans. While the appointment of permanent on-site psychologists and social workers was suggested, participants also recognised the financial constraints facing the public sector. As a result, strengthening existing occupational health services within healthcare facilities to include nursing students may represent a more feasible strategy within South Africa’s constrained economic context.

The findings of this study are particularly important given Lilitha Nursing College’s unique role as the primary public institution responsible for training professional nurses for the provincial health sector in the Eastern Cape. As the province continues to face health workforce shortages and significant service delivery pressures, the experiences of nursing students during the COVID-19 period provide valuable insights for long-term institutional learning. Reflecting on these experiences offers an opportunity to strengthen educational infrastructure, redesign curricula to enhance flexibility, and develop resilient student support systems that can respond to future disruptions. Ultimately, the findings contribute to a broader understanding of how crisis experiences can inform future challenges in nursing education, particularly in resource-constrained settings. By translating students’ lived experiences into institutional learning, policy refinement, and resilience planning, nursing education institutions can better prepare future generations of nurses for complex and uncertain healthcare environments while safeguarding the quality and continuity of training.

**Implications for Nursing Education**

Lessons drawn from Lilitha Public Nursing College students’ experiences during the COVID-19 period offer an opportunity for transformative improvement within nursing education. By investing in digital infrastructure, curriculum innovation, student wellness, and institutional preparedness, nursing education institutions can strengthen their ability to train competent, resilient nurses who can respond to future health system challenges.

**Limitations**

Some limitations should be considered when interpreting the findings of this study. The study was conducted at Lilitha Nursing College in the Eastern Cape Province. Although participants were drawn from multiple campuses, the findings reflect the experiences of students within one public nursing college. A phenomenological design, which typically involves a relatively small number of participants, was used. While this approach provided rich and detailed insights, the sample size may not capture the full range of experiences of all nursing students affected by the pandemic. The findings are based on participants’ personal accounts of their experiences. Self-reported information may be influenced by recall bias, especially when participants reflect on events that occurred earlier during the pandemic. Lastly, the study had limited perspectives, focusing solely on nursing students’ experiences. Perspectives from nurse educators, institutional administrators, and clinical supervisors were not included. Including these stakeholders in future research could provide a more comprehensive understanding of institutional responses to the pandemic and challenges in nursing education.

## 5. Conclusions

This study highlights the profound and interconnected academic, clinical, psychological, and social impacts of COVID-19 on nursing students at Lilitha Nursing College in the Eastern Cape Province. Disruptions to contact teaching, reliance on informal digital platforms such as WhatsApp, digital inequities, and accelerated curricula exposed institutional unpreparedness and contributed to academic overload, poor performance, and delayed programme completion. Students also experienced persistent psychological distress and physical health challenges, while disruptions to clinical training reduced opportunities for skills development and undermined confidence for professional practice. Beyond the immediate pandemic effects, the findings highlight important opportunities for long-term institutional learning within nursing education. They underscore the need to strengthen institutional preparedness through robust digital learning infrastructure, structured academic continuity plans, and coordinated student support systems that can respond effectively during crises. The study also points to the need for curriculum redesign that incorporates flexible teaching approaches, including blended learning, simulation-based training, and the integration of public health emergency preparedness competencies. Furthermore, the findings reveal gaps between educational policy and institutional implementation, particularly regarding mental and physical health support for students. Strengthening integrated wellness services and embedding these within broader health workforce and occupational health frameworks is essential to promote student resilience and academic progression. Overall, the experiences of nursing students during the pandemic provide valuable lessons for strengthening the resilience, quality, and sustainability of nursing education and the future health workforce in South Africa.

## Figures and Tables

**Table 1:** Demographic characteristics of study participants across Lilitha Nursing College campuses.

Code	Gender	Age	Student Level	Facility Name
Participant 1. (P195-1)	Male	48	4th year	Queenstown
Participant 2. (P195-2)	Male	45	4th year	Queenstown
Participant 3. (P195-3)	Male	27	4th year	Queenstown
Participant 4. (P195-4)	Female	40	4th year	Queenstown
Participant 5. (P195-5)	Female	51	4th year	Lusikisiki
Participant 6. (P195-6)	Male	34	4th year	East London
Participant 7. (P195-7)	Female	49	4th year	Queenstown
Participant 8. (P195-8)	Male	28	4th year	Lusikisiki
Participant 9. (P195-9)	Male	45	4th year	Lusikisiki
Participant 10. (P195-10)	Female	45	4th year	Port Elizabeth
Participant 11. (P195-11)	Female	50	4th year	Queenstown
Participant 12. (P195-12)	Female	50	4th year	Lusikisiki
Participant 13. (P195-13)	Female	50	4th year	Queenstown
Participant 14. (P195-14)	Female	37	4th year	Lusikisiki
Participant 15. (P195-15)	Female	51	4th year	Mthatha
Participant 16. (P195-16)	Male	44	4th year	Mthatha
Participant 17. (P195-17)	Female	43	4th year	Mthatha
Participant 18. (P195-18)	Female	28	4th year	Mthatha
Participant 19. (P195-19)	Male	33	4th year	Mthatha
Participant 20. (P195-20)	Female	31	4th year	Mthatha

**Table 2:** Thematic matrix summarizing main themes and sub-themes.

Main Theme	Subthemes	Conceptual Description
Experiences during the COVID-19 pandemic	Disruption to academic progress and learning	Pandemic restrictions disrupted contact teaching, delayed academic schedules, and forced a shift to remote communication platforms such as WhatsApp.
	Psychological and emotional distress	Students experienced fear of infection, anxiety, grief, and emotional strain due to uncertainty, isolation, and exposure to pandemic-related mortality.
	Impact on personal life and social well-being	Lockdown measures altered daily routines, increased family responsibilities, and reduced social interaction and peer support.
Challenges experienced during and post-COVID-19	Mental health challenges	Participants reported ongoing stress, anxiety, depression, and trauma-related symptoms linked to pandemic experiences.
	Physical health challenges	Some students experienced COVID-19 infection and related symptoms, including fatigue, body pain, and breathing difficulties.
	Challenges in clinical and hands-on training	Clinical learning opportunities were disrupted, limiting practical skills development and increasing anxiety about preparedness for practice.
Support services	Institutional support for mental and physical health	Participants reported limited access to counselling services, psychologists, or social workers during the pandemic.
Participants’ recommendations for future preparedness	Strengthening student support systems	Participants recommended improved communication, provision of digital resources, mental health services, and institutional emergency preparedness planning.

## Data Availability

Data supporting reported results is available upon request, a link will be provided.

## Abbreviations

The following abbreviations are used in this manuscript:

SANC	South African Nursing Council
CHE	Council on Higher Education
COVID-19	Coronavirus disease of 2019
LMICS	Low–Middle-Income Countries
